# Tumor radio-sensitivity assessment by means of volume data and magnetic resonance indices measured on prostate tumor bearing rats

**DOI:** 10.1118/1.4941746

**Published:** 2016-02-12

**Authors:** Antonella Belfatto, Derek A. White, Ralph P. Mason, Zhang Zhang, Strahinja Stojadinovic, Guido Baroni, Pietro Cerveri

**Affiliations:** Department of Electronics, Information and Bioengineering, Politecnico di Milano University, Milan 20133, Italy; Department of Radiology, The University of Texas Southwestern Medical Center, Dallas, Texas 75390; Department of Radiation Oncology, The University of Texas Southwestern Medical Center, Dallas, Texas 75390; Department of Electronics, Information and Bioengineering, Politecnico di Milano University, Milan 20133, Italy

**Keywords:** prostate cancer, radio-sensitivity, BOLD, TOLD, mathematical model, LQ model, neural network

## Abstract

****Purpose**:**

Radiation therapy is one of the most common treatments in the fight against prostate cancer, since it is used to control the tumor (early stages), to slow its progression, and even to control pain (metastasis). Although many factors (e.g., tumor oxygenation) are known to influence treatment efficacy, radiotherapy doses and fractionation schedules are often prescribed according to the principle “one-fits-all,” with little personalization. Therefore, the authors aim at predicting the outcome of radiation therapy *a priori* starting from morphologic and functional information to move a step forward in the treatment customization.

****Methods**:**

The authors propose a two-step protocol to predict the effects of radiation therapy on individual basis. First, one macroscopic mathematical model of tumor evolution was trained on tumor volume progression, measured by caliper, of eighteen Dunning R3327-AT1 bearing rats. Nine rats inhaled 100% O_2_ during irradiation (oxy), while the others were allowed to breathe air. Second, a supervised learning of the weight and biases of two feedforward neural networks was performed to predict the radio-sensitivity (target) from the initial volume and oxygenation-related information (inputs) for each rat group (air and oxygen breathing). To this purpose, four MRI-based indices related to blood and tissue oxygenation were computed, namely, the variation of signal intensity $\left(\Delta \mathrm{SI}\right)$ in interleaved blood oxygen level dependent and tissue oxygen level dependent (IBT) sequences as well as changes in longitudinal $\left(\Delta R_{1}\right)$ and transverse $\left(\Delta R_{2}^{*}\right)$ relaxation rates.

****Results**:**

An inverse correlation of the radio-sensitivity parameter, assessed by the model, was found with respect the $\Delta R_{2}^{*}$ (−0.65) for the oxy group. A further subdivision according to positive and negative values of $\Delta R_{2}^{*}$ showed a larger average radio-sensitivity for the oxy rats with $\Delta R_{2}^{*}<0$ and a significant difference in the two distributions (*p* < 0.05). Finally, a leave-one-out procedure yielded a radio-sensitivity error lower than 20% in both neural networks.

****Conclusions**:**

While preliminary, these specific results suggest that subjects affected by the same pathology can benefit differently from the same irradiation modalities and support the usefulness of IBT in discriminating between different responses.

## 1. INTRODUCTION

It is widely acknowledged that the success or failure of prostate cancer radiotherapy depends largely on lesion staging at the time of diagnosis.^1,2^ In spite of a high tumor control probability, especially in early stages, there are degrees of variability in the tumor response (radio-sensitivity) depending on different intrinsic (cell line related) and extrinsic (microenvironment related) factors, which are still under investigation. Among extrinsic factors, tumor oxygenation plays a significant role influencing the radio-sensitivity.^3,4^ For example, hypoxic tumors may require higher radiation doses or different fraction schedules to overcome radioresistance.^5^ Recent preclinical studies have suggested that the tumor radio-sensitivity, for vascularized and well perfused tumors, can be increased by administering hyperoxic gas, inhaled during irradiation.^6–10^ While there is still debate whether tumor oxygenation is a reliable prognostic factor, the assessment of tumor hypoxia allows the treatment tailoring, exploiting the subject-specific radio-sensitivity prediction, which is likely to improve the tumor response.

Oxygen-related data can be gathered by means of various techniques such as a polarographic electrodes (albeit invasive)^11,12^ or positron emission tomography (PET) (requiring the administration of radioactive contrast agents).^13^ By contrast, blood oxygen level dependent (BOLD) and/or tissue oxygen level dependent (TOLD) images, based on functional magnetic resonance techniques, allow a semiquantitative and noninvasive assessment of the average tumor oxygenation.^6,14–16^ In principle, BOLD and TOLD information increases staging reliability and can be exploited to improve the predictive ability of advanced prognostic mathematical tools as well.

In order to predict tumor response to radiation treatment on a patient-specific basis, a large number of *in silico* models have been proposed.^17,18^ Despite the development of hierarchic architectures including the description of phenomena across multiple time and space scales, the lack of their standardized testing and quantitative validation prevents the model translation into clinical practice.^19–21^ Macroscopic approximations, based on the tumor volume regression measured using computed tomography and magnetic resonance imaging, succeeded conversely in predicting tumor response to therapy.^22,23^ In the past decade, the role of oxygenation in tumor growth and responsiveness has been tackled using mathematical models at both macroscopic and microscopic scales.^24–26^

In Ref. 26, we proposed a macroscopic model including interdependent dynamics of tumor evolution and oxygenation based on the following assumptions: (1) the larger the tumor volume, the greater the hypoxic fraction; (2) tumor radio-sensitivity is proportional to oxygenation. At the time, oxygenation could not be assessed and the model validation was based on volume measurements only. In this paper, we refined such a model to represent tumor growth and response to hypofractionated radiotherapy. Our first objective was to verify whether the tumor radio-sensitivity, estimated by such a macroscopic model, correlates with oxygenation indices obtained by interleaved BOLD and TOLD (IBT) MR images (*a posteriori*). The second objective was to verify whether the radio-sensitivity can be assessed at the staging time (*a priori*), exploiting tumor volume and oxygenation data. These objectives were verified by means of experimental procedures performed on eighteen rats implanted with Dunning R3327-AT1 prostate cancer and irradiated using a hypofractionation regimen (subcurative dose).

## 2. MATERIALS AND METHODS

### 2.A. Treatment protocol and data acquisition

This study was approved by the Institutional Animal Care and Use Committee of University of Texas Southwestern Medical Center (protocol 2009-0180). Eighteen male Copenhagen rats were implanted subcutaneously in the thigh with Dunning R3327-AT1 prostate tumor fragments of about 1 mm^3^ from a donor. The tumor size evolution was assessed weekly, by means of a digital caliper, measuring three orthogonal diameters (*a*,  *b*,  *c*). The corresponding volume (*V*) was computed by the spheroid formula $V=\pi /6\left(a,\;b,\;c\right)$.^27^

When the tumor volume reached about 1–2 cm^3^ (mean: 1.2 cm^3^), the eighteen rats were divided into two groups: the air group (*n* = 9) breathed air during irradiation, while the oxy group (*n* = 9) breathed 100% O_2_ for about 15 min, before and during each radiotherapy session.

The day before irradiation, oxygen enhanced MRI was acquired. Rats were anesthetized with 3% isoflurane for induction and maintained with 1.5% isoflurane at 1 l/min. Rats were placed on a plastic bed with a water warming blanket to maintain body temperature. A 35 mm home-built solenoid volume RF coil was used to image the tumors on the thigh of the rats. MR images were obtained using a small animal horizontal bore 4.7 T MR scanner (Agilent Technologies, Palo Alto, CA). Following tumor localization and anatomical imaging (*T*_2_-weighted), images for *R*_1_ were acquired using a sequential variable repetition time (TR) 2-D multislice spin echo sequence (SEMS). Three slices in the center of the tumor featured a thickness of 2 mm, a field of view (FOV) of 60 × 60 mm, an image matrix of 128 × 128 pixels, a TE/TR of 20/100, 200, 300, 500, 700, 900, 1500, 2500, 3500 ms, number of averages of 1, and an acquisition time of 22 min 16 s. IBT images were acquired in two slices using a 2D multislice multiecho spoiled gradient-echo sequence (MGEMS) for BOLD and $R_{2}^{*}$ (thickness = 2 mm, FOV = 60 × 60 mm, matrix=128×128, TE/TR = 6–69/150 ms, echo spacing = 7 ms, flip angle = 20°, number of averages = 3, scan time = 57.6 s) and a 2-D multislice spoiled gradient-echo sequence (GEMS) for TOLD (thickness = 2 mm, FOV = 60 × 60 mm, matrix=128×128, TE/TR = 5/30 ms, flip angle = 45°, number of averages =2, scan time =7.8 s). Interleaved BOLD (*T*_2_*-weighted images) and TOLD (*T*_1_-weighted images) were acquired during baseline air and oxygen for up to 10 min.

**FIG. 1.**
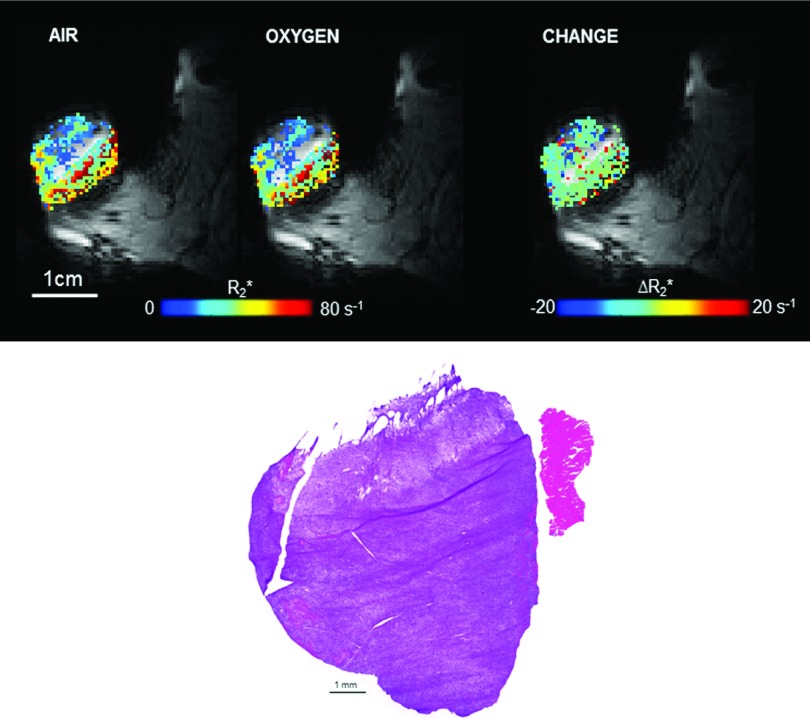
Quantitative $R_{2}^{*}$ maps of a small (1.6 cm^3^) Dunning prostate R3327-AT1 tumor. Upper panel: (Left) Baseline breathing air (mean ± SEM $R_{2}^{*}=42\pm 1\;s^{-1}$), (middle) breathing oxygen (mean ± SEM $R_{2}^{*}=39\pm 1\;s^{-1}$), and (right) $\Delta R_{2}^{*}$ (mean ± SEM = − 3.1 ± 1.4 s^−1^). The $R_{2}^{*}\;\left(1/T_{2}^{*}\right)$ after breathing oxygen had a slower decay compared to air indicative of less deoxyhemoglobin present. Lower panel: After MR imaging, the tumor was resected from the rat thigh and cut in half. Both halves were quickly placed in liquid nitrogen or 10% formalin. The tumor tissue that was fixed in 10% formalin was embedded in paraffin and sectioned for hematoxylin and eosin (H and E) staining. It showed no significant signs of central necrosis, but minimal widespread necroses surrounded by viable tissue.

Four indices of oxygenation were assessed: ΔSI_BOLD_, ΔSI_TOLD_, Δ*R*_1_, and $\Delta R_{2}^{*}$. Percent changes in signal intensity $\left(\Delta \text{SI}\right)$ in BOLD or TOLD response to hyperoxic respiratory challenge were calculated voxel-by-voxel, as $\Delta \text{SI}=100\times \left({\text{SI}}_{\text{oxy}}-{\text{SI}}_{\text{air}}/{\text{SI}}_{\text{air}}\right)$ and averaged over the region of interest. Analysis of BOLD images was based on a single echo time (TE = 20 ms). Local changes in tumor longitudinal relaxation rate (Δ*R*_1_, s^−1^) were calculated voxel-by-voxel as Δ*R*_1_ = *R*_1,oxy_ − *R*_1,air_ and then averaged out. A similar process provided variation of the apparent transverse relaxation rate ($\Delta R_{2}^{*}$, ms^−1^). Representative $R_{2}^{*}$ maps and corresponding histology are shown in Fig. 1.

Postprocessing of MR images was performed offline using in-house algorithms developed in MatLab® (MathWorks, Natick, MA, USA). Table I summarizes the values assessed for each rat.

The next day rats were anesthetized while breathing either air or oxygen and, after an equilibration period of at least 15 min, irradiated. Radiation was delivered using a small animal x-ray irradiator (XRAD 225Cx, Precision X-Ray, North Branford, CT) operating at 225 kV and 13 mA, producing a dose rate of 3.5 Gy/min.

**FIG. 2.**
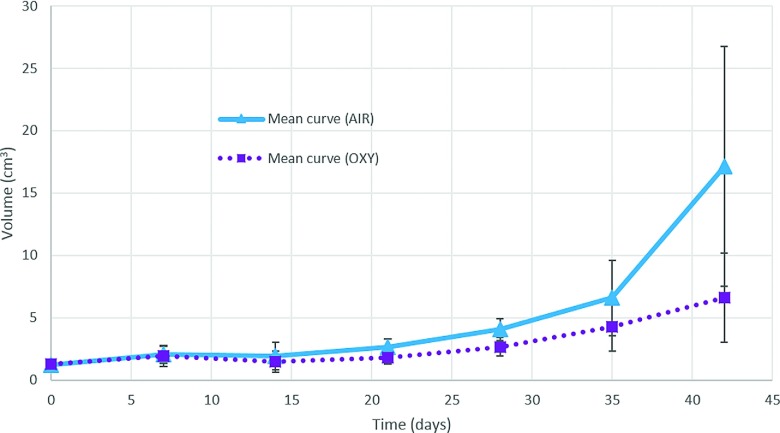
Average evolution curves for the measured tumor volume are shown for air (solid line) and oxy (dotted line) groups separately. Error bars are shown representing ±*σ*, the standard deviation across the sample for the specific time point. Although some rats survived up to 56 days, the plot was stopped at day 42, the last measurement available for all tumors.

Each rat underwent two radiotherapy sessions with two doses of 15 Gy a week apart (30 Gy total). The treatment was planned to be subcurative since the dose (single fraction) at which there is 50% of tumor control probability (TCD_50_) is reported to be about 76 Gy, for the anaplastic AT1 tumor.^28^ Average curves of volume evolution are shown for both groups in Fig. 2.

### 2.B. Mathematical model of tumor evolution

We considered the tumor as consisting of two main regions: (1) a viable (active) volume of clonogens spontaneously growing and affected by radiation therapy and (2) a necrotic volume, not able to proliferate due to either treatment damage or severe hypoxia, which is physiologically washed out. In contrast to previous work,^26^ we did not include an explicit model of tumor reoxygenation along the treatment course or its influence on the radio-sensitivity in the present study.

The modeling of tumor proliferation has been often addressed in the literature.^18,19,21^ Among the most common mathematical formulations for the spontaneous tumor growth, the Gompertzian, Logistic, and exponential equations need to be mentioned.^29,30^ The Gompertzian and Logistic curves feature an initial exponential-like growth that saturates toward an asymptotic value, similar to what is often reported by *in vitro* and *in vivo* studies. In order to achieve this dual behavior, they require the setting of two parameters, namely, the growth rate and the maximum carrying capacity of the tissue. Conversely, despite the potentially unrealistic indefinite growth, the exponential curve can be fully defined by its time constant only. Given the small initial size of the tumors here, and the short observation time window (less than 2 months), a simple exponential function was used. We also assumed, according to the small initial tumor volume, that no necrosis occurs before treatment, as supported by tumor histology (cf., Fig. 1).

At the first irradiation time $\left(t_{\text{ir}1}\right)$, we define $V_{v}\left(t_{ir1}\right)=V\left(t_{\text{ir}1}\right)$ and $V_{n}\left(t_{\text{ir}1}\right)=0$, where *V_v_* and *V_n_* accounts for the active (viable) and necrotic volumes, respectively, while *V* is the overall measured volume. Afterwards, the time evolution of *V_v_* is regulated by the doubling time *T_d_*. *T_d_* refers only to the active volume spontaneous growth and cannot be considered an index of treatment success (growth delay). It reflects the cell-line-specific growth rate and, possibly, environmental factors influencing the cell-cycle and tumor aggressiveness. The radiation therapy effects are usually modeled by means of the linear-quadratic (LQ) model, instead.^23,31^ It defines the surviving fraction $\left(\text{SF}\right)$ as

$$\text{SF}=e^{-\alpha d\left(1-\frac{d}{\alpha /\beta }\right)},$$ (1)

where *d* is the delivered dose and the tumor radio-sensitivity is represented by the *α* (Gy^−1^) and *β* (Gy^−2^) parameters accounting for double (lethal) and single (possibly reparable) strand break damage to DNA, respectively. In order to assess both parameters (*α* and *β*), a study including multiple fractionation strategies would be required (different doses). In order to overcome this issue, we assumed the ratio *α*/*β* = 6.8 Gy according to previous findings on R3327-AT1 rat prostate tumors.^32^ Finally, as the damaged cells are not instantaneously washed out, their dynamics can be described by an exponential decay with an half-time constant *T*_1/2_. In the time between the two irradiation sessions $\left(t_{\text{ir}1}<t\leq t_{\text{ir}2}\right)$, the system can be summarized as

$$V_{v}\left(t\right)=V_{v}\left(t_{\text{ir}1}\right)\text{SF}\;e^{\frac{\ln\left(2\right)}{T_{d}}\left(t-t_{\text{ir}1}\right)}$$ (2a)

$$V_{n}\left(t\right)=V_{v}\left(t_{\text{ir}1}\right)\left(1-\text{SF}\right)e^{-\frac{\ln\left(2\right)}{T_{1/2}}\left(t-t_{\text{ir}1}\right)},$$ (2b)

while for *t* > *t*_ir2_, it can be defined as

$$V_{v}\left(t\right)=V_{v}\left(t_{\text{ir}2}\right)\text{SF}\;e^{\frac{\ln\left(2\right)}{T_{d}}\left(t-t_{\text{ir}2}\right)},$$ (3a)

$$V_{n}\left(t\right)=\left(V_{n}\left(t_{\text{ir}2}\right)+V_{v}\left(t_{\text{ir}2}\right)\left(1-\text{SF}\right)\right)e^{-\frac{\ln\left(2\right)}{T_{1/2}}\left(t-t_{\text{ir}2}\right)}.$$ (3b)

We assumed *α* to be constant throughout time due to the limited amount of data at our disposal and especially the lack of volume measurements between the two irradiations. This assumption, along with the specific fractions applied (same dose delivered at both fractions), allowed us to use the same surviving fraction definition for all the equations above [Eqs. (1)–(3b)]. Similarly to our previous work,^23,26^ the free parameters (*T_d_*, *α*, and *T*_1/2_) were optimized on an animal-specific basis by means of a custom genetic algorithm in order to achieve the best total volume fitting (∀*t*, $V\left(t\right)∼V_{v}\left(t\right)+V_{n}\left(t\right)$). In the parameter learning, *T_d_*, *T*_1/2_, and *α* were bounded in the range 3–7 days, 1–60 days, and 0.005–0.5 Gy^−1^, respectively, according to the prior literature.^22,33^ The large range for *T*_1/2_ was also justified to cope with several dynamics possibly causing a delay in tumor shrinkage (e.g., edema) not explicitly modeled. The fitting error for the *r*th rat $\left(e_{r}\right)$, minimized during the optimization, was computed using the following relation:

$$e_{r}=\frac{\overset{N_{r}}{\underset{i=0}{\sum }}\left|V\left(t_{i}\right)-\left(V_{v}\left(t_{i}\right)+V_{n}\left(t_{i}\right)\right)\right|}{N_{i}},$$ (4)

where *i* identifies each of the *N_r_* time steps at which measured volumes are available. Statistical analysis was performed across the model parameters and the error distributions of the air and oxy groups using the Wilcoxon–Mann–Whitney test (5% significance). The Pearson correlation coefficient (*P*) between the radio-sensitivity parameter (*α*) and the oxygen indices (ΔSI_BOLD_, ΔSI_TOLD_, Δ*R*_1_, and $\Delta R_{2}^{*}$) was computed separately for the oxy and the air groups.

### 2.C. Neural network model

**FIG. 3.**
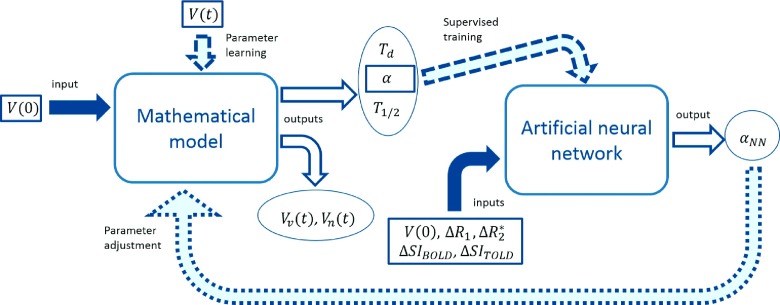
General scheme showing the two main stages of the data processing. First, the mathematical model is trained using all the measured volume obtaining a set of parameters (*T_d_*,  *α*, *T*_1/2_) and the prediction of both viable and necrotic volume evolution for each rat. Second, the radio-sensitivity estimated by the model is used to train a feedforward neural network to predict it according to five inputs, namely, *V*(0), ΔSI_BOLD_, ΔSI_TOLD_, Δ*R*_1_, and $\Delta R_{2}^{*}$. Solid, filled arrows represent inputs, while solid, unfilled and dashed arrows are the outputs and control variables (e.g., target), respectively. Finally, the dotted arrow represents a possible feedback performed employing the *α*_NN_ predicted by the net to set the corresponding parameter of the mathematical model and obtain an estimation of the tumor response of a new rat.

The possibility of an early prediction of tumor radio-sensitivity was investigated using the initial tumor volume and the four indices of oxygen level, namely, the BOLD and TOLD signal intensity variation and the change in longitudinal (*R*_1_) and transverse ($R_{2}^{*}$) relaxation rates. A feedforward artificial neural network (ANN), featuring five input parameters ($V\left(t=0\right),\;\Delta {\text{SI}}_{\text{BOLD}}$, ΔSI_TOLD_, Δ*R*_1_, and $\Delta R_{2}^{*}$), one hidden layer (five neurons), and one output (predicted *α*), was implemented using the built-in Neural Network Toolbox of MatLab® package. For each of the two groups (air and oxy), supervised training was used to estimate the tumor radiation sensitivity using the values provided by the genetic algorithm as targets. A complete scheme of the adopted protocol outlining the two main steps (model fitting and ANN training) is provided in Fig. 3. The prediction ability of the two ANNs was assessed by performing a leave-one-out (LOO) procedure. Out of the nine animals in each group, eight rats were selected for training and one was left out to compute the extrapolation error. This was repeated for all the rats in each group. The prediction error was computed by averaging out all the extrapolation errors.

## 3. RESULTS

### 3.A. Performance of the tumor evolution model

**FIG. 4.**
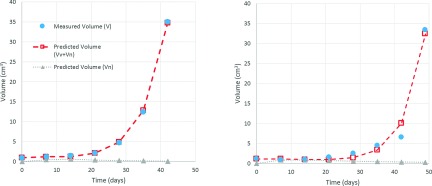
Two examples of fitting curves are reported for a rat belonging to the air group (left panel) and one belonging to the oxy one (right panel). Solid circles and open squares represent the measured and the predicted volumes. Grey triangles (dotted line) stand for the predicted necrotic volume.

The proposed model achieved an average fitting error of about 0.7 cm^3^ (range: 0.2–1.3 cm^3^, 5%–42%) across the eighteen rats. Despite the fact that the error was on average larger for the oxy group (0.8 cm^3^) than that of the air group (0.6 cm^3^), the difference between the two error distributions was not statistically significant (*p* = 0.5) and the model was able to mimic the general volume progression trend in both cases (Fig. 4).

On average, the radio-sensitivity *α* was 0.05 and 0.04 Gy^−1^ for the oxy and air groups, respectively (*p* = 0.27). The assessed tumor doubling time (5.4 and 5.5 days on average for the air and oxy group, respectively) was in accordance with the reported literature.^33^ Finally, the *T*_1/2_ values showed a large variability (11–60 days) and frequent saturation toward the upper bound (Table II). Again, both *T_d_* and *T*_1/2_ distributions were not statistically different across the oxy and air groups.

### 3.B. Radio-sensitivity and oxygenation

**FIG. 5.**
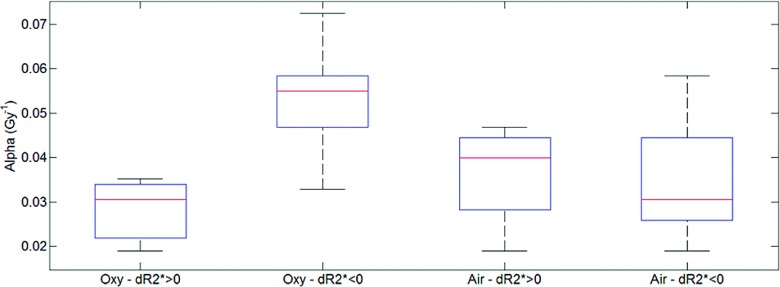
Boxplot of the *α* (alpha) distribution according to the four subgroups identifying numbers of tumors (*n*) in each group (Air_*p*_: air, $\Delta R_{2}^{*}>0$, *n* = 4; Air_*n*_: air, $\Delta R_{2}^{*}<0$, *n* = 5; Oxy_*p*_: oxy, $\Delta R_{2}^{*}>0$, *n* = 3; and Oxy_*n*_: oxy, $\Delta R_{2}^{*}<0$, *n* = 6). The central mark is the median, the edges of the box are the 25th and 75th percentiles, the whiskers extend to the most extreme data points.

A correlation was observed for the radio-sensitivity of the oxygen-breathing rats with respect of ΔSI_BOLD_ (*P* = 0.69) and $\Delta R_{2}^{*}$ (*P* = − 0.65). Both indices are related to the apparent transverse relaxation rate, but given that $R_{2}^{*}$ is a quantitative measurement, we focused on investigating the role of $\Delta R_{2}^{*}$. The two groups were further divided according to their variation of transverse relaxation rate as Air_*p*_ (air, $\Delta R_{2}^{*}>0$), Air_*n*_ (air, $\Delta R_{2}^{*}<0$), Oxy_*p*_ (oxy, $\Delta R_{2}^{*}>0$) and Oxy_*n*_ (oxy, $\Delta R_{2}^{*}<0$). While the Air_*p*_ (*n* = 4) and Air_*n*_ (*n* = 5) subgroups distributions were similar and also comparable to Oxy_*p*_ (*n* = 3), the Oxy_*n*_ set (*n* = 6) featured the largest median value (Fig. 5) and was statistically different from Oxy_*p*_ (*p* < 0.05).

**FIG. 6.**
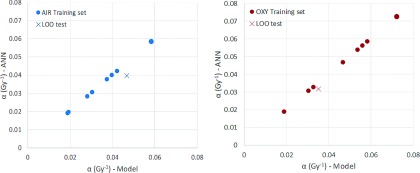
Example of LOO analysis results of the neural network prediction ability for a single item of both air (left panel) and oxy (right panel) groups. Circle and cross markers represent the training data and the test item, respectively.

As expected, fitting accuracy of the ANN (training dataset), in both the oxy and the air groups, was very high (about 100%). The corresponding extrapolation errors, provided by the LOO analysis, were lower than 13% and 19% for oxy and air groups, respectively (Fig. 6).

## 4. DISCUSSION AND CONCLUSIONS

### 4.A. Major findings

The major findings of this work can be summarized as follows: (1) there was a correlation between ΔSI_BOLD_ and *α* (*P* = 0.69), as well as between $\Delta R_{2}^{*}$ and *α* (*P* = − 0.65), in the oxy group; (2) the sign of $\Delta R_{2}^{*}$ distinguished two different distributions of the *α* parameter in the oxy group; (3) the ANN, trained to predict the tumor radio-sensitivity given the initial tumor volume and the MRI indices, yielded an extrapolation error lower than 20% in both groups.

Provided that oxygenation is one of the main microenvironmental factors influencing radio-sensitivity,^4^ a correlation between MRI indices of oxygenation and *α* is expected. The results agree with previous findings in the literature showing a positive correlation between the variation in the BOLD signal response and partial O_2_ pressure (*p*O_2_)^14,15^ and the inverse relation between $R_{2}^{*}$ relaxation rate and the oxygenation level of the tumor.^34^

We investigated possible nonlinear (threshold-like) relations, by means of a further subdivision of the overall dataset into four subgroups according to the $\Delta R_{2}^{*}$ sign. The Air_*n*_ ($\Delta R_{2}^{*}<0$) and Air_*p*_ ($\Delta R_{2}^{*}>0$) subgroups presented a similar distribution of the radio-sensitivity, suggesting that the tumors have comparable microenvironmental conditions. The behavior of the Oxy_*p*_ subgroup, showing radio-sensitivity values in the same range as the Air_*n*_ and Air_*p*_ groups, could be ascribed to a vascularization deficit (immature or defective) which is a quite common consequence of tumor-related fast angiogenesis.^4^ For example, in case of vascular inefficiency, breathing hyperoxic gas may not increase the oxygen level in the region of interest. Conversely, the radio-sensitivity distribution of the Oxy_*n*_ subgroup was significantly different (*p* < 0.05) from the one of Oxy_*p*_ suggesting that a well-vascularized tumor may benefit from oxygen inhalation. It was previously shown that breathing oxygen during a single high dose irradiation could significantly affect the growth of some small Dunning R3327-AT1 prostate tumors.^7,9^ An initial study measured absolute *p*O_2_ directly using ^19^F MRI of the reporter molecule hexafluorobenzene, but this was invasive requiring injection into the tumor.^7^ A later study showed that tumors could be discriminated based on longitudinal relaxation rate response to an oxygen challenge prior to any radiation (ΔSI_TOLD_ and Δ*R*_1_).^9^ Both those studies examined single high dose radiation of 30 Gy, as opposed to the split dose applied here.

The introduction of artificial neural networks to predict the radio-sensitivity at staging time is novel with respect to standard macroscopic model approach.^22–24^ The ANN approach provided prediction accuracy greater than 80% for the radio-sensitivity, showing that it is likely to predict *α* on an individual basis, according to pretreatment data. Despite the fact that the generalization ability of the ANN needs further investigation and would benefit from the inclusion of larger datasets, such a result holds promise.

### 4.B. Model and data issues

Potential shortcomings of the current study can be summarized in (1) small data cohort, (2) measurement precision, (3) lack of quantitative relation between MRI indices and oxygenation, (4) model setting including active and necrotic tumor dynamics, only. The inclusion of only eighteen tumors and the further classification of the animals in two or even four different groups limited the generalization of the work findings. However, we note that the use of a simplified model, featuring three free parameters only, makes it suitable to cope with small data cohorts. Future prospective studies will involve a larger tumor dataset.

The MRI images were acquired only twice during the treatment, namely, the day before each irradiation, but were not used to compute the tumor size. The assessment of the tumor volume was carried out by means of digital caliper measurements, which may have introduced uncertainties we did not quantitatively evaluate. A mismatch between the actual volume and the measured value can be due to inter and intraoperator variability in the diameters measurements, as well as to a nonspheroidal shape of the tumor. However, a standardized protocol^9^ allowed minimizing measurement uncertainty. We plan to address this issue in future studies by an image-based (e.g., MRI) approach to tumor volume assessment. Although the relation between MRI-based indices and oxygenation still remains subject of intense debate, several studies support the positive correlation among oxygenation, ΔSI_BOLD_, ΔSI_TOLD_, and Δ*R*_1_ while $\Delta R_{2}^{*}$ appears inversely correlated.^9,15,34^ In the present study, we were not interested in assessing the absolute tumor oxygenation value per se, but we were rather interested in investigating the relation between oxygen-related indices and the tumor responsiveness to the treatment. It was shown, by means of the neural network approach, that the aforementioned indices can be used, along with the initial volume size, to predict the tumor radio-sensitivity. It has to be remarked that the reference (target) of the neural network is the radio-sensitivity value predicted by the model, which is clearly affected by the limitations in the optimization procedure. Above all, the lack of an independent validation of the two dynamics (active/necrotic) may lead to an incorrect parameter setting. This limitation will be tackled in a future prospective study including multimodal imaging techniques able to provide metabolic information of the tumor (e.g., PET-based).^35^ Finally, some considerations about the simplification of the tumor evolution to two simple macroscopic dynamics (viable and necrotic regions) are in order. First, despite the rough simplification, we were allowed to mimic the tumor growth using an exponential relation due to the early stage of the tumors under investigation, since the leading factor in this case is the uncontrolled cell duplication as stated in the literature.^36,37^ Moreover, the tumor was implanted subcutaneously; therefore, it was not limited in its growth by surrounding structures. Second, there is a debate about whether the LQ formulation is suitable for high dose irradiation.^38,39^ Recent studies suggest that despite being less accurate, the prediction obtained using the LQ is comparable to the one provided by universal survival curves models in case of tumor presenting heterogeneous oxygenation, which applies to most of solid tumors.^40^ This conclusion is further supported in Ref. 41 where the authors argue that the LQ model encompassed a better fit irrespective of treatment doses than did any of the models requiring extra terms at high doses. However, the large error variability (range: 0.2–1.3 cm^3^), as well as the large values of the clearance time constant, suggests that some of the mechanisms that have been discarded were not negligible. For example, irradiation may have triggered a local inflammation, or even vascular damage leading to edema and tumor swelling.^42^ AT1 tumors were indeed found to swell substantially following a single dose of 30 Gy, prior to regression.^7^ Neglecting these dynamics should not impair the overall model parameter estimation, since they usually represent transient states occurring in a short temporal window following the irradiation. This hypothesis is supported by be fact that the error obtained comparing the last measured volume of each rat to the corresponding model approximation was on average less than 5% across the whole dataset. We did not aspire to attain perfect volume fitting, so much as to identify different tumor responsiveness, since large discrepancies in single values could also be due to data noise. Therefore, the three-parameter formulation makes the model more robust and able to mimic the general trend of the volume regression curve despite data uncertainty.

### 4.C. Final remarks

**FIG. 7.**
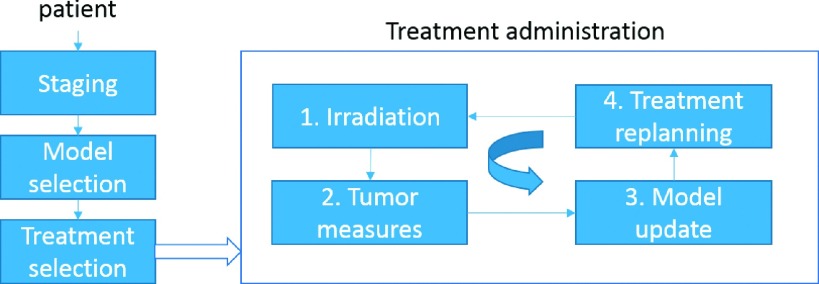
General scheme of treatment planning and adjustment using mathematical models.

In this paper, a macroscopic model of tumor growth and response to radiation therapy was proposed and trained on eighteen Copenhagen rats implanted subcutaneously with Dunning R3327-AT1 prostate cancer and subdivided according to the gas breathed during irradiation (air/oxy). The main goals were to (1) provide an estimation of the individual radio-sensitivity, (2) correlate radio-sensitivity with scalar indices of blood and tissue oxygenation, (3) investigate mathematical methods able to provide an estimation of the tumor responsiveness *a priori*. Despite the limitation of a small dataset, caliper-based measurements and model dynamics reduction, the proposed formulation was able to fit the data within about 25% error in 15 of 18 rats. The correlation analysis suggested a relation between the radio-sensitivity and the changes in the $R_{2}^{*}$ relaxation rate for the oxy group. This hypothesis was supported by further investigation leading to the finding that only rats featuring $\Delta R_{2}^{*}<0$ benefit from oxygen inhalation. In the end, we showed how the radio-sensitivity could be assessed *a priori* using a neural network by means of oxygen and volume-related information. Given that accurate prognosis and radiotherapy personalization are crucial to provide patients with the best possible care,^4,43^ mathematical models able to predict tumor response to different doses and fractionation are gaining popularity. Although further tests and validations are needed to improve their robustness and reliability, we believe that mathematical models of tumor evolution will play a major role in the cancer treatment customization in the near future.^44^ A realistic scenario for the application of mathematical models to therapy personalization is shown in Fig. 7. It encompasses an initial parameter setting (model selection) according to the patient staging using either the literature data or pretreatment information (volume and oxygenation indices). Different radiotherapy modalities (e.g., fractionation and doses) are simulated and the predicted outcome employed to select the one most suitable schedule. A further model refinement can be performed along the treatment administration period based on the differences between prediction and measured volume size along the therapy delivery. This allows for treatment replanning in case of large discrepancies.

## Figures and Tables

**TABLE I.** Characteristics of individual Dunning prostate R3327-AT1 tumors. Inhaled gas during irradiation is shown together with volume at the time of the first irradiation (*V*_0_). The four oxygen-related parameters are presented for each tumor, namely, the variation of signal intensity (BOLD and TOLD) and the change in the relaxation longitudinal and transverse relaxation rates (Δ*R*_1_ and $\Delta R_{2}^{*}$, respectively) caused by oxygen administration during the IBT acquisition.

Rat	Group	*V*_0_ (cm^3^)	ΔSI(%)_BOLD_	ΔSI(%)_TOLD_	$\Delta R_{2}^{*}$ (ms^−1^)	Δ*R*_1_ (s^−1^)
1	Air	1.0	−2.10	−0.40	0.0051	0.0539
2	Oxy	1.5	−0.93	0.32	−0.0010	0.046
3	Air	0.9	3.62	2.13	0.0003	0.0475
4	Oxy	1.1	−3.26	1.07	0.0021	0.1213
5	Air	0.9	−0.60	−0.19	−0.0035	0.0928
6	Oxy	0.7	1.43	0.76	0.0017	0.0393
7	Oxy	0.9	4.52	1.97	−0.0010	0.0677
8	Oxy	1.2	3.55	0.77	−0.0015	0.052
9	Air	1.2	4.09	2.51	−0.0008	0.0374
10	Oxy	1.1	7.94	1.40	−0.0033	0.0308
11	Air	1.2	2.43	0.79	0.0091	0.0149
12	Air	1.4	4.01	1.05	−0.0003	0.0099
13	Air	2.1	−0.44	0.59	−0.0004	−0.0064
14	Oxy	1.6	1.64	1.25	−0.0008	0.031
15	Air	0.9	3.76	2.11	0.0006	0.0365
16	Oxy	1.7	2.60	1.03	0.0054	0.0011
17	Oxy	1.7	4.77	1.51	−0.0012	0.0069
18	Air	1.2	3.29	0.72	−0.0006	0.0472

**TABLE II.** Model parameters and fitting errors obtained by means of a subject-specific optimization.

Rat	Group	*T_d_* (days)	*α* (Gy^−1^)	*T*_1/2_ (days)	*e* (mm^3^)	*e* (%)
1	Air	7.0	0.0189	60	445.67	12.56
2	Oxy	5.4	0.0468	60	692.35	19.11
3	Air	4.0	0.0468	38	1003.48	41.92
4	Oxy	6.8	0.0189	16	382.63	9.23
5	Air	4.8	0.0282	11	191.74	5.11
6	Oxy	6.9	0.0306	60	813.17	23.62
7	Oxy	4.0	0.0584	12	980.49	37.69
8	Oxy	3.5	0.0723	59	1296.24	31.89
9	Air	5.8	0.0305	60	606.78	17.95
10	Oxy	4.1	0.0561	24	1039.49	25.83
11	Air	5.3	0.0375	44	785.70	26.41
12	Air	4.1	0.0584	58	889.99	25.15
13	Air	6.3	0.0398	60	663.06	15.57
14	Oxy	7.0	0.0329	60	542.66	17.28
15	Air	4.1	0.0422	60	394.28	15.70
16	Oxy	7.0	0.0352	60	453.31	15.36
17	Oxy	5.0	0.0538	53	327.90	13.08
18	Air	6.8	0.0189	60	455.51	11.55
